# The Chemical Composition of Endotoxin Isolated from Intestinal Strain of *Desulfovibrio desulfuricans*

**DOI:** 10.1100/2012/647352

**Published:** 2012-05-02

**Authors:** Jolanta Lodowska, Daniel Wolny, Marzena Jaworska-Kik, Sławomir Kurkiewicz, Zofia Dzierżewicz, Ludmiła Węglarz

**Affiliations:** ^1^Department of Biochemistry, Faculty of Pharmacy, Medical University of Silesia, Narcyzow 1 street, 41-200 Sosnowiec, Poland; ^2^Department of Biopharmacy, Faculty of Pharmacy, Medical University of Silesia, Narcyzow 1 street, 41-200 Sosnowiec, Poland; ^3^Department of Instrumental Analysis, Faculty of Pharmacy, Medical University of Silesia, Narcyzow 1 street, 41-200 Sosnowiec, Poland

## Abstract

*Desulfovibrio desulfuricans* anaerobes are constituents of human alimentary tract microflora. There are suggestions that they take part in the pathogenesis of periodontitis and some gastrointestinal inflammatory disorders, such as ulcerative colitis or Crohn's disease. Endotoxin is one of Gram-negative bacteria cellular components that influence these microorganisms pathogenicity. Endotoxin is a lipid-polisaccharide heteropolymer consisting of three elements: lipid A, core oligosaccharide, and O-specific polysaccharide, also called antigen-O. The biological activity of lipopolysaccharide (LPS) is determined by its structure. In this study, we show that rhamnose, fucose, mannose, glucose, galactose, heptose, and 2-keto-3-deoxyoctulosonic acid (Kdo) are constituents of *D. desulfuricans* endotoxin oligosaccharide core and O-antigen. Lipid A of these bacteria LPS is composed of glucosamine disaccharide substituted by 3-acyloxyacyl residues: ester-bound 3-(dodecanoyloxy)tetradecanoic, 3-(hexadecanoyloxy)tetradecanoic acid, and amide-bound 3-(tetradecanoyloxy)tetradecanoic acid.

## 1. Introduction

Bacteria of *Desulfovibrio desulfuricans* species are sulphate-reducing Gram-negative rods settling ecosystems devoid of oxygen. However, *Desulfovibrio* species are capable of growing in the presence of oxygen [1–3]. Abdollahi and Wimpenny [4] stated that some strains of *D. desulfuricans* were able to survive in an aerobic conditions for 24 hours. The tolerance to oxygen can explain the occurrence of *Desulfovibrio* spp. in blood and infected tissues [2]. These commensal bacteria settle mainly in alimentary tract of human and animals. However, they were also isolated from bile tract of patient with symptoms of sepsis [5], abscesses of abdominal cavity, liver, and brain [6–8] and from inflamed appendix [9]. These microorganisms are considered to take part in the pathogenesis of some large intestine disorders such as ulcerative colitis and Crohn's disease [10, 11]. *Desulfovibrio *spp. colonizing the tooth surface is implicated in the etiology of periodontal disease [12, 13].

**Figure 1:**
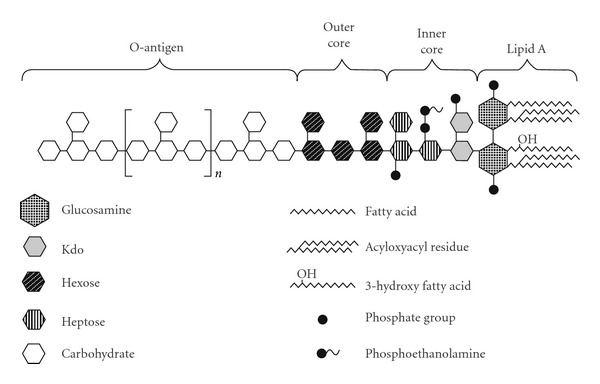
The structure of bacterial endotoxin.

One of the factors responsible for the virulence of Gram-negative bacteria is endotoxin, an integral component of their outer membrane. These polymers are characterized by common structure consisting of three regions: polysaccharide O-antigen, oligosaccharide core, and lipid A (Figure 1). Carbohydrate part of LPS is the most structurally heterogenic region, influencing the bacteria virulence. Its loss may cause the decline of microorganism pathogenicity. O-specific part of LPS consists of repeating oligosaccharide units that are characteristic (in respect of their composition and structure) for a given species of microorganism. These units are commonly built of 2 to 6, and occasionally of 7 or 8, monosaccharides creating straight- or branched-chain oligosaccharide. d-glucose, d-mannose, d-galactose, l-fucose, d- and l-rhamnose are common constituents of O-antigen. Amino sugars in pyranose form such as d-glucosamine, d-mannosamine, d-galactosamine, which may also be N-acetylated (N-acetyl-d-glucosamine and N-acetyl-d-galactosamine) are frequent components of O-antigen. 3,6-dideoxyhexoses can be found in polysaccharide part of *Enterobacteriaceae* endotoxin. Moreover, in the O-specific element of LPS several further carbohydrates were identified, among others: 2-acetamidine-2,6-dideoxy-l-galactose (*Yersinia ruckeri *serotype 01) [14], 3,6-dideoxy-3-(R)-3-hydroxybutyramide-d-galactose (*Proteus penneri *16) [15], N-acetylmuramic acid (*P. penneri *62) [16], and N-acetylneuraminic acid (*Salmonella enterica* serovar Toucra O48*, Hafnia alvei *PCM 2386*, and Escherichia coli *O104) [17, 18].

Another part of LPS is oligosaccharide core consisting of two regions: the outer—built mostly of hexoses and the inner—composed of heptoses and 2-keto-3-deoxyoctulosonic acid (Kdo). 

Kdo links the oligosaccharide core of endotoxin with its hydrophobic part—lipid A, which is composed of d-glucosaminyl-d-glucosamine disaccharide, most commonly substituted by phosphates and ethanolamine or amino-4-deoxy-l-arabinose at positions 1 and 4′, and at positions 2, 3, 2′ and 3′ by hydroxy fatty acids. (R)-3-hydroxy fatty acids (most often (R)-3-hydroxytetradecanoic acid) are usually amide-bound to the carbohydrate core of lipid A, whereas (S)-2- or (R)-3-hydroxy fatty acids are ester-bound to glucosamine disaccharide. Hydroxy fatty acids can be acylated by other fatty acids [19]. Glucosamine is not an obvious constituent of carbohydrate core of lipid A. Some bacteria for example, *Pseudomonas diminuta* and *P. vesicularis* have this core made of two molecules of 2,3-diamino-2,3-dideoxy-d-glucose linked by the *β*(1→6) glycosidic bond [20]. However, this structural modification does not contradict the greatest structural conservatism of lipid A as compared to other LPS parts.

The structure and chemical composition of *D. desulfuricans* LPS are not fully recognized that justifies the effort to elucidate them.

## 2. Material and Methods

### 2.1. Bacterial Strain and Culture Conditions

The wild strain DV/A of *D. desulfuricans* was isolated from the feces of patient with asiderotic anemia and cholestasis as described previously [21] and cultured under anaerobic conditions in a Postgate's liquid medium containing pyruvate [22].

### 2.2. Isolation of Bacterial Lipopolysaccharide

LPS was isolated from the cultured bacteria using the method of Westphal et al. [23] and Shynra et al. [24]. Briefly, suspension of bacterial cells was deproteinized with phenol at 68°C. Aqueous phase, separated by centrifugation and prepurified by dialysis, was incubated in the presence of DNase, RNase, magnesium sulfate, and chloroform. The mixture was again purified by dialysis, once against acetate buffer (pH 5) and three times against water. After adjusting of pH to 8 with Tris buffer, the solution was incubated with proteinase K and dialyzed against water.

### 2.3. Analysis of Core and O-Antigen Composition

For determining carbohydrate profile of the core and the O-specific region, hydrolysis with 1% acetic acid (2 h, 100°C) was used to separate polysaccharide fragment of endotoxin from the lipid A which precipitated during centrifugation at 3400 ×g [25]. The supernatant was evaporated in a stream of argon, and the remaining sugar constituents were prereduced with aqueous-methanolic solution of NaBD_4_ [25]. The oligosaccharide derivative was hydrolyzed with 2 M trifluoroacetic acid (TFA) at 121°C for 2 h [26]. After evaporation, monosaccharides were reduced to alditols with NaBD_4_ in 1 M ammonia (10 mg/mL) at 4°C for 12 h [26, 27]. The excess of reducing agent was removed with glacial acetic acid, and the borates formed were evaporated in a stream of argon. Then, three portions of 10% acetic acid in methanol followed by one portion of methanol alone were added, and the solvent was evaporated in a stream of argon every time. The obtained alditols were N-acetylated by incubation in the presence of acetic anhydride and saturated solution of sodium bicarbonate at room temperature for 30 min [26]. O-acetylation of the alditols was performed by incubation of the sample with acetic anhydride at 120°C for 20 min in the presence of sodium acetate as a catalyst [28]. The reaction mixture was then evaporated in the presence of toluene, washed with dichloromethane, and reevaporated. The alditol acetates were extracted with hexane/water mixture (1 : 1, v/v) and analyzed by GC/MS technique.

The analyses were performed with the use of a Hewlett Packard 5890 series II gas chromatograph interfaced to a Hewlett Packard 5989A mass spectrometer. The carbohydrate derivatives were separated on a RESTEK RTX-5MS fused silica capillary column (5% diphenyl, 95% dimethylpolysiloxane, and 60 m × 0.32 mm i.d.; film thickness: 0.25 *μ*m). The GC oven temperature was programmed from 100°C (isothermal for 1 min) to 180°C at a rate of 20°C/min, then to 250°C at a rate of 3°C/min. The final temperature was held for 12 min. Helium was used as a carrier gas at a flow rate of 1.8 mL/min, and the GC column outlet was connected directly to the ion source of a mass spectrometer. The GC/MS interface was kept at 280°C, while the ion source and the quadrupole analyzer were maintained at 200°C and 100°C, respectively.

### 2.4. GC/MS Identification of Kdo and GlcN in Examined LPS

LPS isolated from *D. desulfuricans *was methanolyzed for 1 h at 100°C with 2 M methanolic hydrochloric acid (0.5 mL), obtained in the reaction of acetyl chloride with anhydrous methanol. The sample was evaporated under the stream of argon, and methyl glycosides were acetylated at 100°C by 30 min with acetic anhydride (100 *μ*L) in the presence of pyridine (100 *μ*L). Acetylation reagents were removed under a stream of argon, and sample was washed twice with methanol and applied on GC/MS [29]. Analysis was performed on the same apparatus as in case of carbohydrates, which was equipped with HP-1MS capillary column (60 m—length, 0.32 mm—internal diameter, 0.25 *μ*m—film), at the programmed temperature: 50°C initial temperature for 1 min, raised to 160°C with 20°/min and then raised to 260°C with 4°/min. Separated products were analyzed by Hewlett Packard HP 5989A mass spectrometer. Ionization was performed by 70 eV electron impact (ion source temperature—200°C, quadrupole—100°C).

### 2.5. Determination of Lipid A Fatty Acids and 3-Acyloxyacyl Residues

Ester-, amide-bound total fatty acids, and 3-acyloxyacyl residues present in *D. desulfuricans* lipid A were analyzed by a gas chromatography coupled with mass spectrometry (GC/MS) according to the procedures described by Wollenweber and Rietschel [19]. To determine the total fatty acid profile, endotoxin was treated with HCl followed by methanolic HCl, and the obtained methyl esters were analyzed by GC/MS.

Ester-bound fatty acids were selectively liberated from endotoxin by methanolic NaOCH_3_ as the corresponding methyl esters and subjected to GC/MS analysis. Treatment of LPS with methanolic NaOCH_3_ yielded de-O-acetylated LPS containing amide-bound fatty acids that were analyzed using the procedure for total fatty acid determination [19].

To determine 3-acyloxyacyl residues, lipid A was prepared by mild acid hydrolysis of LPS (1% acetic acid; 1 h; 100°C), and amide-bound 3-acyloxyacyl residues were liberated from it in the form of their methyl esters after conversion to acid-labile imidate by methyl iodide in the presence of silver salts. Ester-bound 3-acyloxyacyl residues were liberated as well, due to the presence of trace amount of water. Therefore, to distinguish between ester- and amide-bound compounds, the procedure was conducted once in the presence and once in the absence of methyl iodide.

The analyses of fatty acid and 3-acyloxyacyl residues were carried out on Hewlett-Packard gas chromatograph (HP5890 II) coupled with mass spectrometer (HP5989A). Capillary column HP5-MS (60 m; i.d. 0.32 mm; film 0.25 *μ*m; Agilent Technologies) was used. Samples of total, ester- and amide-bound fatty acid methyl esters were injected on a column at 60°C in the splitless mode. The oven temperature was programmed from 60°C to 100°C at 10°C/min, then at 5°C/min up to 260°C which was maintained for 13 min. The samples of 3-acyloxyacyl derivatives were injected on a column at 50°C in the splitless mode. The oven temperature was programmed from 50°C to 160°C at 20°C/min, then at 3°C/min up to 290°C which was maintained for 25 min.

### 2.6. UV-Vis Determination of 2-Keto-3-deoxyoctonic Acid (Kdo), Glucosamine (GlcN), and Phosphate Groups

The presence of Kdo in endotoxin was verified spectrophotometrically by the thiobarbituric acid method [30]. LPS was hydrolyzed with sulphuric (VI) acid at 100°C for 30 min to liberate Kdo. Then, Kdo was subjected to react successively with periodic acid, sodium arsenite (III), and thiobarbituric acid to form a chromophore with absorbance maximum at 550 nm.

Hexosamine was analyzed by spectrophotometry according to the Elson-Morgan method [31]. LPS was incubated with 2 M TFA for 1 h at 120°C. Next, the sample was evaporated under the stream of nitrogen, water, and basic acetylacetone (obtained by mixing of acetylacetone with sodium carbonate) were added, and mixture was incubated for 15 min at 100°C. Afterwards, *p*-aminobenzaldehyde (ADAB) solution (4 g ADAB dissolved in 300 mL of HCl mixed with ethanol; 1 : 5; v : v) was added, and incubation was continued for 30 min at 75°C. After cooling, the absorbance was measured at 512 nm using Hewlett-Packard type 8452A spectrophotometer.

Phosphate content in LPS was evaluated by the Bartlett's method [32]. LPS was dissolved in the deionized water, 5M sulfuric (VI) acid was added, and sample was incubated for 3 h at 150–160°C. 30% H_2_O_2 _was then added, and incubation was continued at 150–160°C for 1.5 h. After cooling, 0.22% ammonium molybdate and Fiske-SubbaRow reagent were added, and the reaction mixture was heated on the water bath. The absorbance was measured at 820 nm.

## 3. Results

### 3.1. Carbohydrates of Core and O-Antigen of Endotoxin

**Figure 2:**
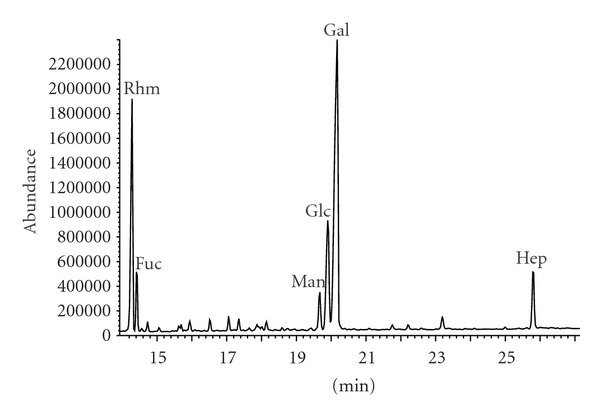
Chromatogram of alditol acetates derived from the core and O-antigen of *D. desulfuricans* endotoxin.

**Figure 3:**
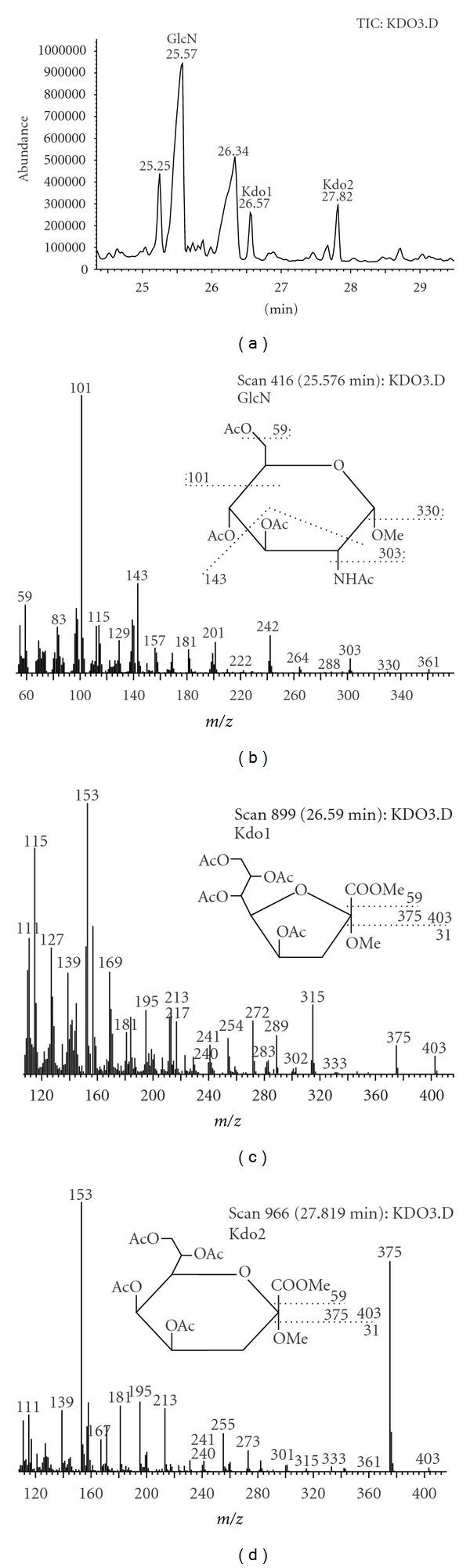
Chromatogram and mass spectra of glucosamine and Kdo methyl glycoside originated from LPS of *D. desulfuricans*.

The interpretation of chromatogram (Figure 2) and mass spectra of alditol acetates revealed rhamnose, fucose, mannose, glucose, galactose, and heptose as the components of polysaccharide chain of *D. desulfuricans* LPS. Galactose and rhamnose were predominant carbohydrates in LPS structure, accounting for 45.3% and 23.3% of all identified sugars, respectively (Table 1). The percentage of fucose was five times lower than rhamnose. The content of mannose was also low (4.2%), whereas glucose amounted to 16.4% in carbohydrate profile. Moreover, the derivatization of carbohydrates of the investigated endotoxin to acetylated methyl glycosides showed that Kdo and glucosamine were also present in its structure (Figure 3). The quantity of Kdo, Glc and phosphate groups per 1 mg of investigated LPS is presented in Table 2.

### 3.2. Total Fatty Acids

**Figure 4:**
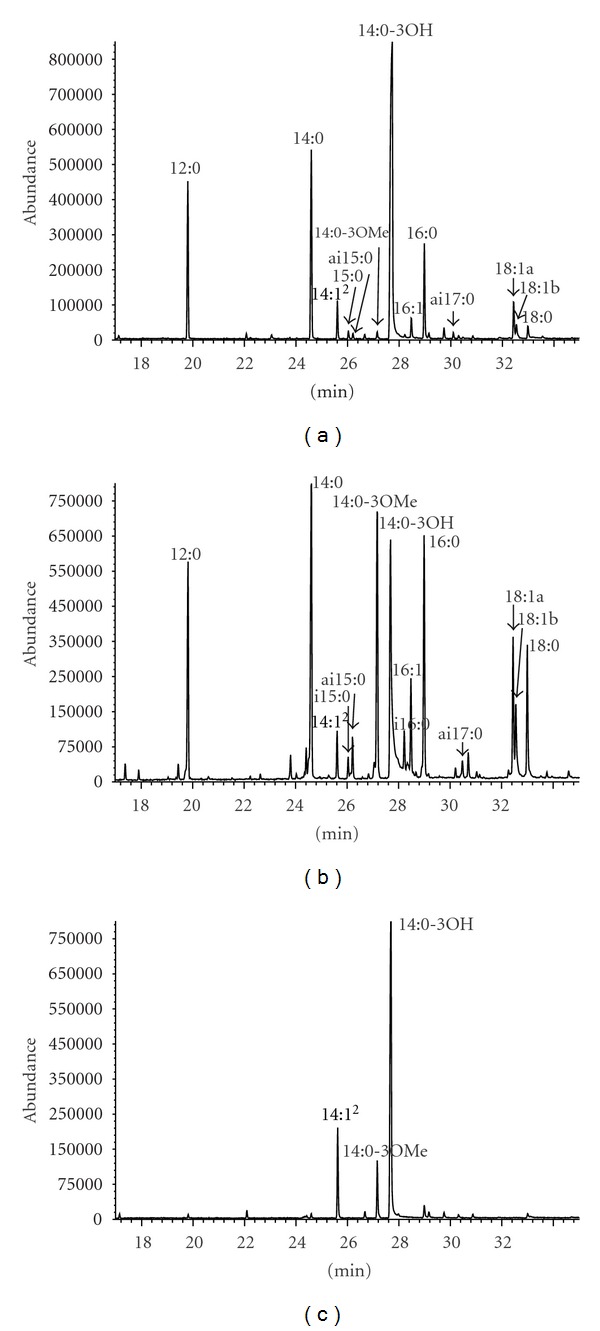
Chromatogram of methyl derivatives of fatty acids being the constituents of *D. desulfuricans* LPS (a) total fatty acids, (b) ester-bound fatty acids, (c) amide-bound fatty acids).

The GC/MS analysis showed fatty acids of the C12–C18 chain length in *D. desulfuricans* endotoxin (Table 3 and Figure 4(a)). The predominant fatty acid was 3-hydroxytetradecanoic acid (3-OH 14:0). Its derivatization produced not only its methyl ester but also methyl esters of 3-metoxytetradecanoic acid (3-OMe 14:0) and tetradecenoic acid (14:1). Furthermore, dodecanoic (12:0), tetradecanoic (14:0) and hexadecanoic acid (16:0) methyl esters were found in significant amounts among analyzed compounds. The other fatty acid derivatives were detected in smaller amounts.

### 3.3. Ester- and Amide-Bound Fatty Acids

The fatty acid analysis revealed that 12:0; 14:0; 3-OH 14:0 and 16:0 were ester-bound in lipid A, as peaks of their derivatives predominated on the obtained chromatograms (Figure 4(b)). The peak of 3-OMe 14:0 acid methyl ester was derived from 3-OH 14:0 substituted at its hydroxy group by other fatty acid, following its reaction with methanolic NaOCH_3_. Therefore, it can be suggested that this fatty acid was a component of ester-bound 3-acyloxyacyl residue. Other low-intensity peaks probably originated, as it was also observed in the total fatty acid analysis, from contamination of LPS extracts by membrane lipids. The GC/MS analysis also showed that the only amide-bound fatty acid in the investigated endotoxin was 3-OH 14:0 (Figure 4(c)). The peaks of 14:1 and 3-OMe 14:0 derivatives, seen on chromatograms, are artifacts formed from 3-OH 14:0 fatty acid during derivatization procedure.

### 3.4. 3-Acyloxyacyl Residues of Lipid A

**Figure 5:**
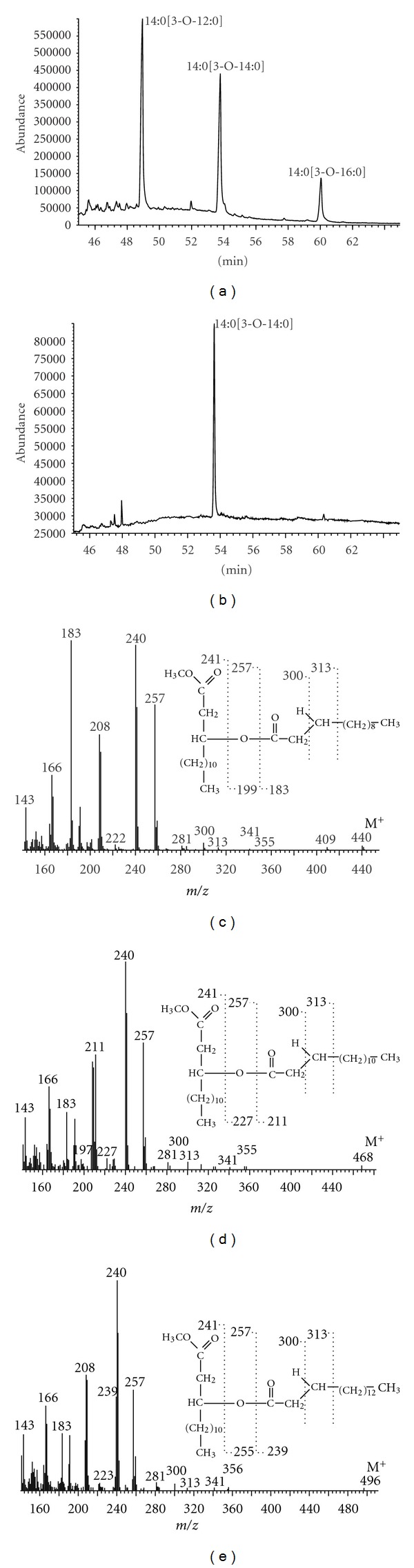
Chromatograms and mass spectra of 3-acyloxyacyl ligands of carbohydrate core of *D. desulfuricans* lipid A (chromatogram of derivatives obtained in the presence (a) and in absence (b) of methyl iodide; mass spectrum of 3-(dodecanoyloxy)tetradecanoic (c), 3-(tetradecanoyloxy)tetradecanoic (d), and 3-(hexadecanoyloxy)tetradecanoic (e) methyl esters).

3-(dodecanoyloxy)tetradecanoic, 3-(tetradecanoyloxy)tetradecanoic, and 3-(hexadecanoyloxy)tetradecanoic acids were identified in lipid A of *D. desulfuricans* DV/A strain (Figure 5(a)). The derivatization procedure conducted in the absence of CH_3_I indicated that 3-(tetradecanoyloxy)tetradecanoic acid was ester-linked to glucosamine core of lipid A, whereas the two other 3-acyloxyacyls were amide-bound to it (Figure 5(b)). Due to the lack of 3-acyloxyacyl methyl ester spectra in the mass spectra database (Wiley 7), the identification of these compounds was performed by the comparison with theoretical fragmentation and with spectra found in other papers [33–35]. The obtained mass spectra of 3-acyloxyacyl methyl esters are shown in Figure 5. As a characteristic for each of these residues, molecular ions (M^+^) at *m*/*z* = 440, 468, and 496 were used as a major criterion of their identification. Ions at *m*/*z* = 241 and 257, observed on every spectrum, indicate 3-hydroxytetradecanoic acid, whereas substituents of 3-OH 14:0 were identified by the presence of ions at *m*/*z* = 183 (CH_3_(CH_2_)_10_CO), 211 (CH_3_(CH_2_)_12_CO), and 239 (CH_3_(CH_2_)_14_CO) on the respective mass spectra (Figures 5(c)–5(e)).

## 4. Discussion

Endotoxin is an immunogen stimulating the immune system cells to liberate the inflammatory mediators, which can cause pathophysiological effects such as septic shock, leukopenia, leukocytosis, activation of complement, hyperglycemia, lowering of blood pressure, and Shwartzman reaction.

Endotoxin of *D. desulfuricans* DV/A strain had no stimulatory effect on epithelial colon cells Caco-2 [36]. These cells did not release IL-8 after stimulation with DV/A endotoxin; however following incubation with LPS and sodium butyrate, the increase of IL-8 synthesis was observed [37, 38]. Furthermore, the treatment of Caco-2 cells for 1 h with increasing concentrations of this LPS (10, 50, and 100 *μ*g/mL) resulted in the decrease of IL-6 and IL-6 receptor genes expression, whereas elongation of treatment with 50 *μ*g/mL of LPS to 6 h increased transcriptional activity of both genes [39]. *D. desulfuricans* endotoxin enhanced the secretion of IL-6 and IL-8, and induced the expression of adhesion molecules—selectin E and VCAM-1 (vascular adhesion molecule-1) in vascular endothelial cells, what indicates that it influences the expression of genes-encoding proteins involved in inflammatory processes [37]. LPS of the investigated bacteria in the concentration of 30 *μ*g/mL decreased metabolic activity of V-79 fibroblasts, inhibited their growth and caused the apoptosis of these cells, indicating its influence on cell proliferation [40]. Dzierżewicz et al. [41] found that endotoxin of *D. desulfuricans* DV/A strain in concentration of 100 *μ*g/mL inhibited the growth of human gingival fibroblasts HGF-1, whereas at its lower concentrations this effect was not observed. Moreover, *D. desulfuricans* endotoxin enhanced the secretion of IL-6 and IL-8 by HGF-1 cells and showed the ability to induce TNF*α* synthesis by mononuclear blood cells [21].

The above-mentioned biological activity of *D. desulfuricans* endotoxin is determined by its chemical structure. The structure of lipid A, the center of LPS toxicity, influences the immunomodulatory properties of endotoxin. Wolny et al. [42] proved that two molecules of glucosamine, probably linked by the *β*(1→6) glycosidic bond, were the constituents of carbohydrate core of lipid A. The structurally similar lipid A core is synthesized by *Escherichia coli *[43], *Moraxella catarrhalis *[44], *Proteus mirabilis *[45], *Neisseria meningitidis *[46], *Rhodobacter sphaeroides* [47], and many other bacteria. In the present study, 3-(dodecanoyloxy)tetradecanoic and 3-(tetradecanoyloxy)tetradecanoic residues were found to be amide-bound and 3-(hexadecanoyloxy)tetradecanoic residue was ester-bound to the disaccharide core of *D. desulfuricans* DV/A lipid A, which means that dodecanoic, tetradecanoic, hexadecanoic, and 3-hydroxytetradecanoic acids are components of the investigated LPS. This observation is congruent with findings of Wolny et al. [42] concerning the fatty acid profile of several other *D. desulfuricans* strains. These fatty acids could also be found in lipid A of *Escherichia coli *[43], *Proteus mirabilis *[45], *Haemophilus influenzae *[48], *Neisseria meningitidis *[46], *Salmonella typhimurium* [49], and so forth.

Among derivatives of *D. desulfuricans* LPS fatty acids, besides just mentioned 3-OH 14:0; 12:0; 14:0 and 16:0, small amounts of branched (i15:0, ai15:0, i16:0 and ai17:0), unsaturated (16 : 1 and 18 : 1) and 18 : 0 fatty acids were also identified. Probably, these fatty acids are not components of investigated strain lipid A and their presence in analyzed samples is a result of their strong association with lipopolysaccharides. These fatty acids may be constituents of cellular membrane lipids because they were identified as a cellular fatty acids in *D. desulfuricans* [50]. Edlund et al. [51] identified different fatty acids with chain length of 14 to 19 carbon atoms, both straight and branched, saturated, and unsaturated with diversified location of double bonds in lipopolysaccharide of *D. desulfuricans*. While, Gaylarde and Beech [52], investigating *D. desulfuricans* LPS chemical composition, identified atypical for such structures fatty acids—8-octadecenoic and tetracosanoic acid. They demonstrated also 9-octadecenoic, 10-octadecenoic, heptadecenoics, and eicosenoic acids, but no hydroxy fatty acids in lipid A of these bacteria.

The high biological activity of lipid A is caused by the presence of six, asymmetrically located fatty acids and also by the presence of additional substituents, for example, phosphate groups. Rietschel et al. [53] claim that the fewer phosphate groups occur in lipid A the less it is active. The lack of one of the phosphate substituents results in 100 times lower biological activity of LPS. Lipid A of *D. desulfuricans* is phosphorylated the thing that influences its activity.

The chemical structure of many microorganisms, for example, *Proteus penneri* 71 [54], *Proteus penneri* 63 [55], *Pseudomonas syringae* pv. *garcae *ICMP 8047 [56], *Yersinia enterocolitica* serotype O:28 [57], and *Vibrio cholerae* H11 [58] has been elucidated so far; however, there is little know about the structure of carbohydrate part of *D. desulfuricans* LPS. Gaylarde and Beech [52] only attempted to determine the carbohydrate profile of these bacteria endotoxin. They identified rhamnose, glucose, galactose, mannose, and ribose. These findings are considerably similar to the results presented in this paper because all these sugars, except ribose, were detected. The presence of ribose in the investigated LPS is questionable, since Gaylarde and Beech [52] themselves admitted that this carbohydrate originated from nucleic acids being the impurities of the sample.

Rhamnose was found in the analyzed endotoxin. This 6-deoxyhexose commonly occurs in lipopolysaccharides. It was detected in LPS of *Salmonella arizonae* O62 [59], *S. enteritidis* [60], *Shigella dysenteriae* [61], *Pseudomonas syringae* pv. *ribicola* NCPPB 1010 [62], and pv. *garcae* ICMP 8047 [56]. There was also other methylopentose-fucose and three hexoses-mannose, glucose, and galactose—in the polysaccharide chain of the investigated strain of *D. desulfuricans.* Hexoses may be constituents of not only O-antigen but also the outer part of the oligosaccharide core, whereas, heptose is component of the inner part of the core. In endotoxin of many microorganisms, the heptose part of oligosaccharide core is linked with Kdo, which in turn is linked with lipid A. Gaylarde and Beech [52] suggested the lack of Kdo in *D. desulfuricans* LPS. The GC/MS analysis of alditol acetates obtained after derivatization of DV/A strain seemed to prove the hypothesis of Gaylarde and Beech because the derivative of Kdo was not identified. However, since Kdo is a labile compound, decomposing in the conditions of derivatization to the alditol acetates [63, 64], the presence of this sugar in *D. desulfuricans* LPS was verified by its analysis in the form of acetylated glycosides. This allowed confirming that this eight-carbon ketose with carboxylic group is a component of investigated LPS. The UV-Vis determination of this sugar allowed establishing that the quantity of Kdo per 1 mg of LPS was 25.8 *μ*g. According to Lee and Tsai [65], the thiobarbituric acid method cannot be used when Kdo is substituted at position 4 or 5, which prevents its oxidation by periodic acid. The investigation of Carof et al. [66], concerning the structure of *Bordetella pertussis* endotoxin, also proved that Kdo with phosphate group or phosphoethanolamine at position 4 cannot be determined by thiobarbituric acid method. Thus, the results showed in this paper suggest that Kdo, present in *D. desulfuricans* endotoxin, has no substituents at position 4 or 5. It should be pointed out that 3,6-dideoxyhexose—abequose and colitose—can also react with thiobarbituric acid [67]; however, these carbohydrates are rare in bacterial endotoxins except *Enterobacteriaceae,* and the GC/MS analysis did not show their presence.

## Figures and Tables

**Table 1:** Quantities of carbohydrates in polysaccharide chain of *D. desulfuricans *endotoxin.

	Rhamnose	Fucose	Mannose	Glucose	Galactose	Heptos**e**
%*AUP* ± *SD*	23.3 ± 2.1	5.0 ± 0.5	4.2 ± 0.5	16.4 ± 2.5	45.3 ± 5.4	5.8 ± 0.3

%AUP: percentage of area under the peak of carbohydrate in the carbohydrate profile of core and O-antigen.

SD: standard deviation.

**Table 2:** The quantity of Kdo and GlcN i phosphates (PO_4_) in LPS of *D. desulfuricans* intestinal strain (DV/A).

	m (*μ*g/mg LPS)	
Kdo	GlcN	PO_4_
25.8 ± 0.5	23.1 ± 2.7	12.6 ± 0.4

**Table 3:** Fatty acid profile of *D. desulfuricans *endotoxin.

	%AUP ± SD
12:0	12.1 ± 0.09
14:0	16.1 ± 0.1
15:0	0.6 ± 0.06
ai15:0	0.4 ± 0.03
3OH 14:0	53.2 ± 0.16
16:1	1.7 ± 0.11
16:0	7.1 ± 0.14
ai17:0	0.6 ± 0.25
18:1a	3.1 ± 0.15
18:1b	1.7 ± 0.08
18:0	1.1 ± 0.09

%AUP: percentage of area under the peak of fatty acid in the fatty acid profile of *D. desulfuricans *endotoxin.

SD: standard deviation.
